# The impact of the COVID-19 pandemic on neurosurgery in the elderly population in Sweden

**DOI:** 10.1186/s12889-024-18332-0

**Published:** 2024-03-15

**Authors:** Michael Axenhus, Sophia Schedin-Weiss, Lars Tjernberg, Bengt Winblad

**Affiliations:** 1 Department of Clinical Sciences at Danderyd Hospital, Karolinska Institutet, Stockholm, Sweden; 2 Division of Neurogeriatrics, Department of Neurobiology, Care Sciences and Society, Karolinska Institutet, Stockholm, Sweden; 3 Theme Inflammation and Aging, Karolinska University hospital, Huddinge, Sweden

**Keywords:** COVID-19, Neurosurgery, Public health, Surgery, Sweden

## Abstract

**Background:**

The COVID-19 pandemic prompted a refocus of health care resources to acute care which has impacted on the capacity of healthcare systems to conduct neurological surgeries. The elderly population has been shown to be particularly vulnerable to the consequences of the pandemic. Less neurosurgery can result in great impact on public health by increasing morbidity and mortality in patients with malignancies and traumatic injuries. The aim of this study was to investigate the effects of the COVID-19 pandemic on neurosurgical procedures in the elderly population in Sweden.

**Methods:**

In this retrospective observational study, the reported incidence of all neurosurgical procedures registered in the 21 Regions of Sweden during 2015–2021 in people aged 65 year or older was collected. Surgical procedures were classified according to the NOMESCO system of classification. Neurosurgery incidence was defined as the number of NOMESCO associated interventions per 100.000 inhabitants. ICD-10 codes associated with neurosurgery-related diagnoses and deaths were also collected. Expected incidence of neurosurgery, neurosurgery-associated deaths and brain cancer diagnoses was estimated and compared to actual outcomes. Decrease in the incidence of neurosurgery was compared to regional COVID-19 incidence, other types of surgery and surgery waiting times.

**Results:**

The incidence of several categories of neurosurgery decreased in Sweden during 2020 and 2021, although not as much as other surgical categories. Women were more affected than men by the decrease in neurosurgery which could be partly explained by a decrease in brain cancer diagnoses amongst women. There was an association between regional decrease in neurosurgery incidence and longer surgery waiting time. COVID-19 incidence in the region did not have an effect on regional decreases in neurosurgery incidence.

**Conclusions:**

The COVID-19 pandemic resulted in a reduction in the number of neurosurgical procedures performed in Sweden during 2020–2021, although not as much as in other European countries. There was regional difference in Sweden with respect to number of surgeries, and waiting time for elective surgeries although there was no increase in mortality.

**Supplementary Information:**

The online version contains supplementary material available at 10.1186/s12889-024-18332-0.

## Introduction

The SARS-CoV-19 (COVID-19) pandemic has forced healthcare providers to refocus care efforts in order to prioritize acute care for COVID-19 patients and patients with serious medical conditions [1, 2]. This has had significant impact on public health around the world with forecasting modellings showing cumulative case numbers with almost exponential growth and mortality rates of non-communicable diseases that might exceed the mortality caused by COVID-19 [3, 4]. The reorganization of healthcare resources to meet the COVID-19 related disease burden, has led to consequences for other areas of healthcare. In particular, care requiring complex diagnostics and invasive procedures, such as surgery for malignancy, has suffered delays [5, 6]. Changes in management of oncology cases require restructuring entire clinical pathways across wide regional networks [7]. Surgeries have been reported to be postponed within several fields and changes in mortality trends for tumors have been reported [8, 9]. The elderly population has been severely affected by the pandemic with high mortality rates and the secondary effects of the COVID-19 pandemic on the elderly population are not fully elucidated [10].

The large number of COVID-19 patients and their associated care has interfered with the availability of staff, material, and equipment necessary to perform surgical procedures. Infections amongst healthcare staff and relocation to other healthcare sectors have also affected the ability to perform surgery during the pandemic [11]. There has been a significant backlog of required surgeries, in particular amongst elective surgical procedures [12, 13]. Although surgical focus has been directed at patients with potential life threating conditions, the effects of the COVID-19 pandemic on neurosurgical procedures amongst the elderly is only partially evaluated. Insights into the effect of the COVID-19 pandemic on specific areas of surgery amongst the elderly is lacking as well as the impact on diagnostic procedures [14].

Sweden held a unique position during the pandemic. Lockdowns did not occur, mask mandates did not exist, and overall mobility was not as restricted as in other European countries. These factors might have influenced neurosurgical care. The purpose of this study was to evaluate neurosurgery-related healthcare in Sweden amongst the elderly during the COVID-19 pandemic compared to pre-pandemic years and the effect of the pandemic on mortality rate in common neurosurgical indications, number of brain cancer diagnoses and surgery waiting times. By examining the incidence of neurosurgical procedures during the pandemic, knowledge about the most affected areas of neurosurgery can be gained and help guide future healthcare strategies.

## Materials and methods

### Data acquisition

Sweden is a high-income country with 10 million inhabitants as of 2022. The first wave of COVID-19 in Sweden occurred in April–July 2020, a second wave followed in October 2020–February 2021, and a third wave in February–May 2021. The population of Sweden was 10,352,965 in July 2020, 10,392,954 in February 2021 and 10,486,251 in May 2021 (Table 1) [15].

**Table 1:** Total number of men and women living in Sweden during the three waves of the pandemic

	Jul-20	Feb-21	May-21
**Men**	5,222,847	5,225,917	5,234,335
**Women**	5,130,118	5,167,037	5,251,916
**Total**	10,352,965	10,392,954	10,486,251

Sweden consists of 21 administrative Regions that are responsible for the collection of healthcare data. Regional healthcare data is reported to a national agency, the National Board of Health and Welfare, for quality assurance and research purposes. Surgeries and surgery related interventions are classified and registered according to the Nordica Medico-Statistical Committee (NOMESCO) classification system [16].

We defined neurosurgery incidence as the number of registered neurosurgical NOMESCO interventions per 100.000 inhabitants. We collected the incidence of neurosurgical procedures amongst people aged 65 years or older according to NOMESCO (Categories A-AW), smaller neurosurgical interventions (Category TA), and diagnostic procedures within neurosurgery (Category XA) in Sweden from 2015 to 2021 from the National Board of Health and Welfare. We specifically chose to study surgery incidence starting at 2015 and onwards in order to emphasize recent trends. The NOMESCO data was quality checked by cross-referencing it to the known number of neurosurgeries performed in the Stockholm Region. This data was obtained by internal review of surgery records.

We also obtained regional population level data to determine the ratio of surgical procedures per 100.000 inhabitants. Regional and gender specific differences in neurosurgery incidence were also investigated. ICD-10 codes used for the calculation of expected incidence of diagnoses and mortality rates of malign, benign or unknown tumors, as well as hydrocephalus (C69–72, D32–33, G91) were obtained from the same source [17, 18]. COVID-19 incidence data was obtained from the Public Health Agency of Sweden [19]. Waiting times for neurosurgery in 2021 were obtained from the Swedish Association of Local Authorities and Regions. Surgery waiting times during 2020 were unavailable [20].

All data was anonymized, publicly available and therefore not subjected to ethical review.

### Statistics

We used NOMESCO reporting of surgery from 2015 to 2019 to estimate the expected incidence in 2020–2021 and compared expected versus actual outcome.

Logistic regression models with the surgery procedural coding rate as the dependent variable and year as a continuous independent variable were used to estimate the yearly change in surgery incidence between 2015 and 2019. The expected incidence of surgical procedures for 2020 and 2021 was calculated using the line formula derived from the logistic regression models. We calculated expected mortality per ICD-10 code in the same manner by replacing the dependent variable with the mortality rate of each ICD-10 code. We assumed the expected incidence of surgeries and mortality per ICD-10 code to be the same for 2020 and 2021. Neurosurgery incidence was compared to other surgeries in order to compare differences between various types of surgery. Regional neurosurgery incidence was correlated to average monthly waiting times for neurosurgery to determine if neurosurgery incidence affected waiting times. Actual outcome that differed from expected outcome by at least 2 standard deviations was considered significant. Two-tailed t-test was used to test for significance with *p* < 0.05 being considered significant. Pearson correlation test was used to determine association where appropriate. Data analysis was performed in SPSS version 28.0 (IBM, USA). All graphs were created using GraphPad version 9.4.0 and edited in Adobe Illustrator version 27.0.

## Results

We first calculated the expected incidence of neurosurgery per 100.000 inhabitants and compared to actual outcome for 2020. All categories but one showed a significant decrease in incidence during 2020; of note, 5 categories out of 8, as well as the cumulative incidence of neurosurgery per 100.000 inhabitants showed a statistically significant decrease between 2019 and 2020 (Table 2).

**Table 2:** Differences in neurosurgery incidence per category per 100.000 inhabitants during 2020

Category	2019	2020	Expected value 2020 versus 2020 (95% CI)	P
**Surgeries on the nervous system**	**734.8**	**589.3**	**0.82(0.79–0.86)**	<0.001
Surgeries on the skull and intracranial structures	209,4	192.4	0.96(0.91–1.01)	ns
Surgeries on the spinal cord and nerve roots	248.4	189.9	0.76(0.72–0.80)	<0.001
Surgeries on peripheral nerves	267.7	196.8	0.60(0.58–0.62)	<0.001
Surgeries on the autonomic nervous system	0.1	0.1	0.96(0.90–1.02)	ns
Various surgeries on the nervous system	16.2	15.2	0.89(0.84–0.94)	<0.001
Reoperations on the nervous system	7.8	6.4	0.85(0.80–0.90)	<0.001
Smaller neurosurgical interventions	417.5	367.4	0.92(0.88–0.96)	0.0093
Diagnostic procedures within neurosurgery	62.8	45.3	0.76(0.68–0.84)	<0.001

Quota value is expected incidence over actual outcome

We then compared neurosurgery incidence to other types of surgeries. Three categories of surgery showed a larger decrease in incidence than neurosurgery. Surgical categories that showed no change in outcome compared to expected value were surgery on the breast, surgery on the pleura and mediastinum, obstetric surgery, surgery on the aorta, peripheral and lymphatic vessels, obstetric surgery and smaller surgical interventions (Table 3).

**Table 3:** Surgical categories and corresponding changes in incidence during 2020

Surgical category	Expected value 2020 versus 2020 (95% CI)	P
Smaller surgical interventions	1.00(0.99–1.01)	ns
Surgeries on the aorta, peripheral vessels and lymphatic system	1.00(0.98–1.02)	ns
Obstetric surgery	1.01(0.96–1.06)	ns
Surgeries on the breast, pleura, and mediastinum	0.99(0.97–1.01)	ns
Surgeries on the skin	0.91(0.86–0.96)	0.003
Surgery on endocrine organs	0.90(0.87–0.93)	<0.001
Surgeries on the nervous system	0.82(0.79–0.86)	<0.001
Surgeries on the gastrointestinal tract and associated organs	0.91(0.89–0.93)	<0.001
Transluminal endoscopy	0.90(0.87–0.93)	<0.001
Surgeries on the peripheral vessels and the lymphatic system	0.81(0.78–0.84)	<0.001
Surgeries on the heart and large intrathoracic vessels	0.93 (0.90–0.96)	0.015
Surgeries on the urinary tract, male genitals and the tissue behind the peritoneum	0.89(0.86–0.92)	<0.001
Surgeries on the female genitals	0.89(0.85–0.93)	<0.001
Diagnostics in connection with surgical activities	0.88(0.82–0.94)	<0.001
Breast gland surgeries	0.86(0.83–0.089)	<0.001
Surgeries on the musculoskeletal system	0.81(0.78–0.84)	<0.001
Surgeries on the ear, nose, throat and trachea	0.84(0.77–0.93)	<0.001
Surgeries on the lips, teeth, jaws, mouth and pharynx	0,85(0.79–0.92)	<0.001
Surgeries and special examinations in the eye region	0.91(0.86–0.96)	0.021
Removal of organ for the purpose of transplantation	0.72(0.57–0.87)	<0.001

Quota value is expected incidence over actual outcome

Categorizing the data according to gender showed a significantly larger decrease in neurosurgery incidence amongst both men and women aged 65 years or older with women experiencing a slightly larger decrease (*p* < 0.001). There were no differences between men and women in certain subcategories of neurosurgery, primarily elective surgery such as spinal surgery, surgery on peripheral nerves, and diagnostic procedures (Table 4).

**Table 4:** Sex differences in neurosurgery incidence in Sweden during 2020

Category	Sex	2019	2020	Expected value 2020 versus 2020 (95% CI)	P
**Surgeries on the nervous system**	**Men**	**778.4**	**644.9**	**0.85(0.80–0.90)**	**<0.001**
**Surgeries on the nervous system**	**Women**	**696.9**	**540.9**	**0.79(0.74–0.84)**	**<0.001**
Surgeries on the skull and intracranial structures	Men	249.3	228.4	0.95(0.89–1.01)	ns
Surgeries on the skull and intracranial structures	Women	174.7	160.9	0.97(0.92–1.04)	ns
Surgeries on the spinal cord and nerve roots	Men	259.7	205.7	0.79(0.72–0.86)	<0.001
Surgeries on the spinal cord and nerve roots	Women	238.5	176.1	0.73(0.67–0.79)	<0.001
Surgeries on peripheral nerves	Men	257.4	200.4	0.82(0.75–0.89)	<0.001
Surgeries on peripheral nerves	Women	276.6	193.7	0.72(0.66–0.78)	<0.001
Surgeries on the autonomic nervous system	Men	0.3	0.1	0.63(0.49–0.90)	0.035
Surgeries on the autonomic nervous system	Women	0.0	0.1	0.94(0.80–1.14)	ns
Various surgeries on the nervous system	Men	19.3	16.9	0.83(0.77–0.89)	<0.001
Various surgeries on the nervous system	Women	13.6	13.7	0.96(0.91–1.01)	ns
Reoperations on the nervous system	Men	9.5	8.6	0.88(0.78–0.98)	0.038
Reoperations on the nervous system	Women	6.2	4.5	0.81(0.76–0.86)	<0.001
Smaller neurosurgical interventions	Men	473.4	431.8	0.97(0.92–1.03)	ns
Smaller neurosurgical interventions	Women	369.0	311.1	0.86(0.76–0.96)	0.014
Diagnostic procedures within neurosurgery	Men	55.2	41.1	0.77(0.65–0.89)	<0.001
Diagnostic procedures within neurosurgery	Women	69.4	49.0	0.76(0.65–0.87)	<0.001

Quota value is expected incidence over actual outcome. Incidence is per 100.000 inhabitants

Fewer diagnoses might result in decreased neurosurgery incidence. Hence, we examined how many neurosurgery-related diagnoses were registered during 2020 and 2021 and compared this to previous years. There were fewer diagnoses of malign tumors amongst women during 2021, but not during 2020, compared to previous years (Table 5).

**Table 5:** Changes in neurosurgery associated diagnoses amongst men and women 2018–2021

ICD-10 code	Sex	2018	2019	2020	2021	Expected value 2020 versus 2020 (95% CI)	P
C71-Malign tumor in the brain	Men	34.3	32.2	32.8	34.2	0.94(0.97–1.01)	ns
C71-Malign tumor in the brain	Women	24.2	25.2	23.9	21.3	0.96(0.92–1.00)	ns
D43-Tumor of unknown origin in the brain and other parts of the central nervous system	Men	30.9	33.7	29.3	31.3	0.88(0.81–0.95)	ns
D43-Tumor of unknown origin in the brain and other parts of the central nervous system	Women	28.6	26.0	24.0	24.1	0.96(0.91–1.01)	ns

Quota value is expected incidence over actual outcome. Incidence is per 100.000 inhabitants

Next, we investigated whether improved conditions and lower pandemic load on healthcare systems might result in more surgeries during 2021 compared to 2020. We found no evidence of an increased incidence of neurosurgery during 2021. There were tendencies to increases in some categories, although these were not significant (Table 6).

**Table 6:** Difference in neurosurgery incidence per category during 2021

Category	2020	2021	Expected value 2021 versus 2021 (95% CI)	P
**Surgeries on the nervous system**	**589.3**	**660.8**	0.91(0.86–0.96)	<0.001
Surgeries on the skull and intracranial structures	192.4	212.3	1.04(0.98–1.10)	ns
Surgeries on the spinal cord and nerve roots	189.9	205.8	0.80(0.74–0.87)	<0.001
Surgeries on Peripheral Nerves	196.8	233.1	0.87(0.83–0.92)	<0.001
Surgeries on the autonomic nervous system	0.1	0.1	1.00(0.98–1.02)	ns
Various surgeries on the nervous system	15.2	16.1	0.95(0.91–1.01)	ns
Reoperations on the nervous system	6.4	6.2	0.84(0.82–0.86)	<0.001
Smaller neurosurgical interventions	367.4	405.4	0.97(0.91–1.02)	ns
Diagnostic procedures within neurosurgery	45.3	36.6	0.62(0.56–0.68)	<0.001

Quota value is expected incidence over actual outcome. Incidence is per 100.000 inhabitants

A significant portion of neurosurgeries are performed at the 6 largest university hospitals in Sweden. We compared the number of surgeries performed in each region with a university hospital to look for regional differences. Only two Regions reported the expected number of neurosurgical procedures during 2020 and 2021. One Region showed no decrease in 2020 but a subsequent decrease in 2021 (Table 7).

**Table 7:** Incidence of neurosurgery in Swedish Regions during 2020 and 2021

Region	Expected 2020 value versus 2020 (95% CI)	P	Expected 2021 value versus 2021 (95% CI)	P
Stockholm	1,02(0.99–1.05)	ns	0,99(0,97–1.01)	ns
Uppsala	1,03(0,98-1,08)	ns	0,99(0,96–1.02)	ns
Östergötland	0,87(0,84-0,90)	<0,001	0,81(0,75-0,87)	<0,001
Skåne	0,86(0,81-0,91)	<0,001	0,85(0,80–0.90)	<0,001
Västra Götaland	0,91(0,88-0,94)	0,011	0,75(0,72-0,78)	<0,001
Västerbotten	1,02(0,98-1,06)	ns	0,84(0,81-0,87)	<0,001

Quota value is expected incidence over actual outcome

At this point, we also performed a quality control of the data by cross-referencing the data obtained from the National Board of Health and Welfare with internal surgery records of the Stockholm region. The difference between the reported incidence and internal reporting was < 2% with local records reporting more surgeries than official statistics, possibly due to inconsistencies in reporting and surgery coding classifications (Supplemental Table 1).

**Fig. 1:**
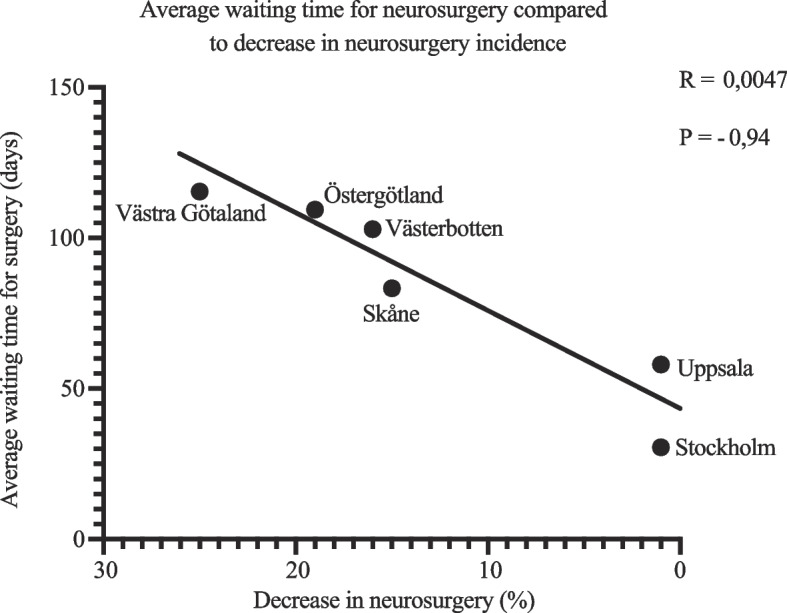
Average regional waiting time for neurosurgery compared to decreases in regional neurosurgical incidence in six Swedish regions during 2021. Decrease in neurosurgery incidence is represented as change from expected value in percentage

The average waiting time for neurosurgery in six Swedish regions during 2021 was collected and correlated to regional changes in neurosurgery incidence. A decrease in neurosurgery incidence was associated with longer waiting times for neurosurgery, *p* = 0.0047. Neurosurgery waiting times for 2020 were unavailable (Fig. 1).

Fewer neurosurgeries might result in increased mortality in common indications for neurosurgery. We compared the mortality of malign and benign tumors in the brain and spine (ICD-10 codes C69–72, D32–33) and hydrocephalus (ICD-10 code G91) during 2020 to previous years. There were no changes in mortality for either tumors or hydrocephalus, indicating that a decrease in neurosurgery incidence did not result in higher mortality rate in common neurosurgical indications amongst people aged 65 years or older during 2020 (Table 8) or 2021 (Supplementary Table 2).

**Table 8:** Mortality rate in common indications for neurosurgery

ICD-10 code	2019	2020	Expected value 2020 versus 2020 (95% CI)	P
C69 Malignant tumor of the eye and nearby tissues	1.03	1.07	1.12(1.02–1.18)	ns
C70 Malignant tumor of the membranes of the central nervous system	0.10	0.13	1.07(0.87–1.24)	ns
C71 Malignant tumor of the brain	16.72	17.80	0.97(0.88–1.08)	ns
C72 Malignant tumor of the spinal cord, cranial nerves and other parts of the central nervous system	0.04	0.10	0.89(0.76–1.02)	ns
D32 Benign tumor of the membranes of the central nervous system	2.65	2.93	1.18(0.99–1.47)	ns
D33 Benign tumor of the brain and other parts of the central nervous system	0.14	0.15	1.09(0.87–1.24)	ns
G91 Hydrocephalus	0.97	1.37	1.20(1.02–1.45)	ns

Quota value is expected incidence over actual outcome. Incidence is per 100.000 inhabitants

## Discussion

In this study we explored the number of all neurosurgical procedures performed in Sweden during the height of the COVID-19 pandemic during 2020–2021 amongst people aged 65 years or older, and compared neurosurgery to other types of surgery, waiting times for surgery, and mortality. We found that the number of neurosurgical procedures decreased during the pandemic in both 2020 and 2021. This decrease correlated to longer waiting time for surgery, while there was no impact on mortality in neurosurgical indications. These results have implications for future neurosurgical care as the rationing of care was a relevant ethical-legal threat to neurosurgical practice over the first phases of the pandemic in many European countries [21].

Our study showed that neurosurgery in Sweden appears to be less affected by the COVID-19 pandemic compared to other countries. A study examining non-elective neurosurgery in Austria and The Czech Republic found a 13% decrease in neurosurgery incidence along with no change in mortality rates [22]. A study of neurosurgery incidence in Ireland saw a 9% decrease in the number of neurosurgeries performed [23]. Smaller studies from Germany, Spain, and the US have reported changes in neurosurgery incidence ranging from 16 to 58% depending on the time frame and neurosurgery subtype studied [24–26].

We found the incidence of elective subtypes of neurosurgery to be decreased. Predictions made before the height of the pandemic stated that the amount of elective surgeries were expected to decrease significantly [27]. Many studies have reported a decreased number of non-imperative surgeries such as elective spine surgery, pain surgeries and functional neurosurgery [28, 29]. Given the overall decrease in surgeries it is not unexpected that reoperations also decreased.

As regional COVD-19 incidence was not associated with decreases in neurosurgery incidence, alternative explanations must be considered. Availability of staff and access to standardized testing could differ between Regions. Patient presentation is also an important aspect as patients were less inclined to seek healthcare during the pandemic [30, 31]. Women in particular were more likely to perceive the pandemic as a serious healthcare concern and therefore comply with public policy measures [32]. Fewer diagnoses of brain tumors amongst women could affect neurosurgery incidence. Longer waiting times for neurosurgery might influence future mortality rate in neurosurgical indications. Further investigation into regional handling of neurosurgical care during the pandemic might provide explanations for differences in neurosurgery incidence.

Neurosurgery interest groups, such as the European Association of Neurosurgical Societies, were early in recognizing the difficulties faced by surgery clinics during the COVID-19 pandemic and position statements were quickly produced [33, 34]. The quick implementation of new guidelines and protocols might have been beneficial for the neurosurgical patient population. Although the correlation is uncertain, studies that have evaluated mortality trends in neurosurgical indications have found mortality trends to be mostly unchanged despite fewer surgeries, indicating proper triage [25, 26]. Furthermore, the rapid adoption of new technology is likely a significant factor in reducing mortality and improving performance amongst neurosurgical units [35].

Except for the effect on the patient population, physician training also needs to be considered. Many neurosurgery residents have reported a negative impact on their training due to fewer surgeries during the COVID-19 pandemic [36, 37]. Some strategies might be implemented to help neurosurgery residents. For example, telemedicine has been introduced as a viable alternative to outpatient consultations [38]. The use of standardized protocols and precautionary measures could improve surgery rates [39].

There are weaknesses inherent in this data: it is retrospective, and Regions might report COVID-19 incidence unevenly. There was also an inconsistency of about 1–2% between official neurosurgery incidence and local surgery records.

In conclusion, the impact of the COVID-19 pandemic on neurosurgery incidence in Sweden was not as extensive as has been reported in other countries. There were regional variances in neurosurgery incidence which could not be explained using the current datasets. There were longer waiting times for neurosurgery although no effect on the mortality rate in neurosurgical indications. Women were more affected than men by the changes in neurosurgicalc care. Decreased neurosurgical care have implications for both physician training and patient populations. Clinicians should be aware of potential future complications due to longer waiting times and decreased surgery volumes.

### Supplementary Information

**Supplementary Material 1.**

**Supplementary Material 2.**

## Data Availability

All data is available from the corresponding author on reasonable request.

## Abbreviations

COVID-19: SARS-CoV-19
NOMESCO: Nordica Medico-Statistical Committee
